# Hemorrhagic shock caused by preoperative computed tomography-guided microcoil localization of lung nodules: a case report

**DOI:** 10.1186/s12893-022-01696-8

**Published:** 2022-06-27

**Authors:** Fan Yang, Jie Min

**Affiliations:** Department of Radiology, The Second Affiliated Hospital, Zhejiang University School of Medicine, 88 Jiefang Road, Shangcheng District, Hangzhou, 310009 Zhejiang China

**Keywords:** Microcoil localization, Delayed hemopneumothorax, Video-assisted thoracoscopic surgery, Hemorrhagic shock

## Abstract

**Background:**

Video-assisted thoracoscopic surgery (VATS) is an emerging technology in minimally invasive surgery, which has become recognized as standard treatments for early-stage lung cancer. Microcoil localization is considered to be a safe and effective way of preoperative localization, and is essential to facilitate VATS wedge-resection for lung nodules.

**Case presentation:**

Here we report a rare case of a 28-year-old female who developed hemorrhagic shock caused by delayed pneumothorax after preoperative computed tomography (CT)-guided microcoil localization. The thoracic CT revealed hydropneumothorax in the right thoracic cavity at 10 h after microcoil localization, and the patient later had significant decreased hemoglobin level (87 g/L). Emergency thoracoscopic exploration demonstrated that the hemorrhagic shock was induced by delayed pneumothorax, which led to the fracture of an adhesive pleura cord and an aberrant vessel. Electrocoagulation hemostasis was then performed for the fractured vessel and the patient gradually recovered from the hypovolemic shock.

**Conclusions:**

Microcoil localization is a relatively safe and effective way of preoperative localization of lung nodules, however, hemorrhagic shock could be induced by rupture of pleural aberrant vessels subsequent to puncture related pneumothorax. Shorten the time interval between localization and thoracoscopic surgery, extend the monitoring time after localization might help to reduce the risk of these complications.

## Background

Lung cancer is one of the most prevalent cancer worldwide, which has a lower 5-year survival rate (~ 29.7%) than that of other malignant tumors (~ 58.6%), due to that patients are often diagnosed at late stages of cancer [1]. With wide application of low-dose chest high resolution computed tomography (CT) screening, the discovery rate of early-stage lung cancer is increasing in recent years [2]. Early detection paves the way for in-time intervention, which provides better outcomes for patients. Recently, minimally invasive video-assisted thoracosopic surgery (VATS) has become the mainstream choice for early-stage lung cancer [3]. However, wedge resection under VATS for ground glass opacity nodules (GGN) is challenging, because the small nodules are difficult to locate the nodule with tactile sensation or naked eyes. Therefore, the precise preoperative localization of the GGN is of great importance to thoracoscopic surgery, which could shorten the operation time and improve the accuracy of resection. Among all the localization methods, microcoil localization has the lowest total complication rate because of the structural characteristics and mechanical properties of a microcoil [4, 5]. It is composed of platinum and has a thrombogenic fiber coating, which could help in reducing the possibility of procedure-related complications [6, 7]. Here we report a rare case with a severe delayed hemopneumothorax following the microcoil localization and its management, reminding us to re-examine the safety of microcoil localization. The criteria of CT-guided microcoil localization for GGNs before VATS are as follows: (1) GGNs that persisted for more than 3 months; (2) GGNs with diameter of less than 3 cm; (3) the distance from the GGNs to the pleura was less than 4 cm; (4) GGNs with suspected malignancy; and (5) GGNs that were invisible and impalpable during VATS.

## Case presentation

**Fig. 1:**
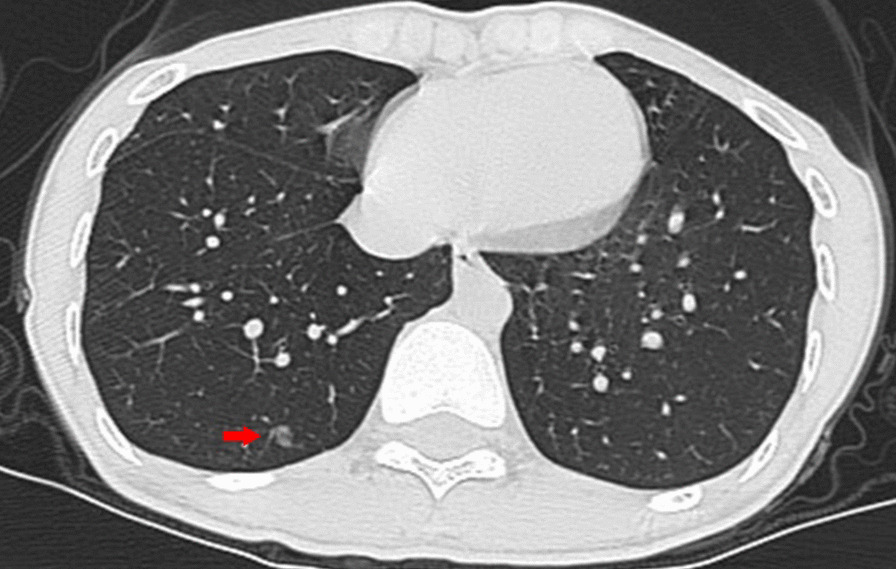
Plain chest CT image shows a ground glass opacity nodule (red arrow) in the right lower lobe of the lung

**Fig. 2:**
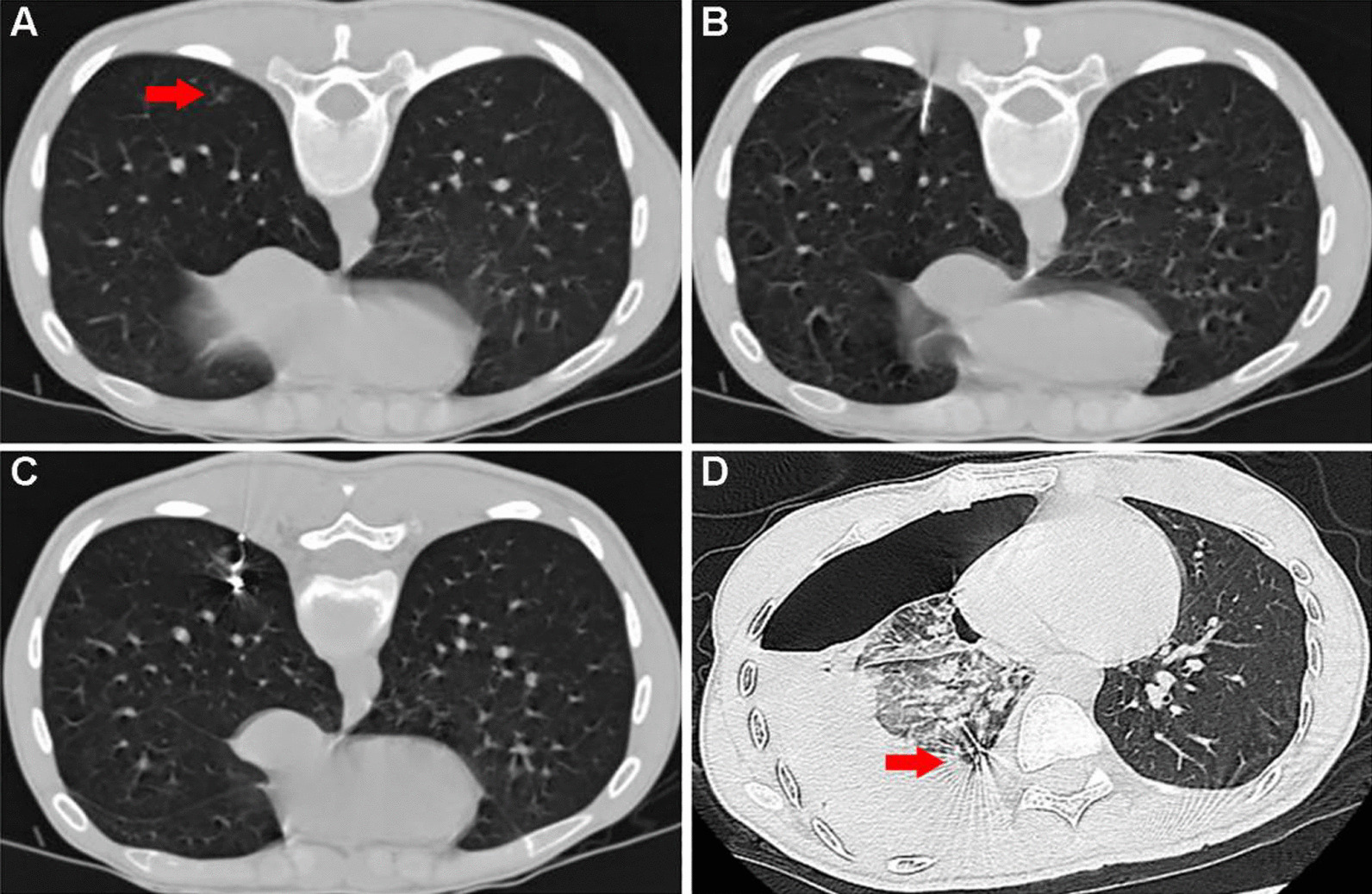
**A** The location of the ground glass nodule (red arrow) in prone position. **B** The tip of the introducer needle was placed adjacent to the edge of the nodule. **C** The distal end of microcoil was placed next to the nodule and the proximal end was left on the parietal pleura. No obvious pneumothorax was seen. **D** Emergency chest CT reveals massive hydropneumothorax and right lung collapse at 10 h after the localization. The microcoil can still be found in the lower right lung (red arrow)

A 28-year-old female presented to a pulmonologist for evaluation of a GGN, which was found in the posterior basal segment of the right lower lobe. The patient had no history of fever, weight loss, shortness of breath, chest pain or other chest complaints. In fact, a GGN with the size of 6 × 4 mm was detected 2 years ago. The size of the GGN increased gradually in the follow-up chest CTs (Fig. 1), and surgical resection of the nodule was recommended by the department of thoracic surgery. The patient underwent CT-guided percutaneous pneumocentesis for positioning 1 day prior to surgery at our radiology department, and had opted for local anesthesia with 2% lidocaine after CT localization (Fig. 2A). The procedures were as follows: the CT-guided percutaneous puncture was carried out using the introducer needle (Argon Medical Devices Inc., Athens, TX 75751, USA), which preloaded with the microcoil (Cook incorporated, Bloomington, IN 47,404, USA). When the needle tip reached (but not touching) the edge of the nodule (Fig. 2B), a 5-mm-long microcoil was pushed out into the lung, allowing it to mark the location of the nodule. The needle was then retracted into the pleural space and pulled out until the proximal end of the microcoil was deployed at the edge of the lung. The CT scan was subsequently performed to ensure the right position of the microcoil (Fig. 2C), which showed one end of the coil anchored along the nodule and the other in the pleural space. No obvious pneumothorax or hematoma was demonstrated. The patient had no apparent clinical manifestations, such as pain, hemoptysis, chest distress, or shortness of breath, and was monitored for heart rate, blood pressure and blood oxygen for the ensuing 4 h.

**Fig. 3:**
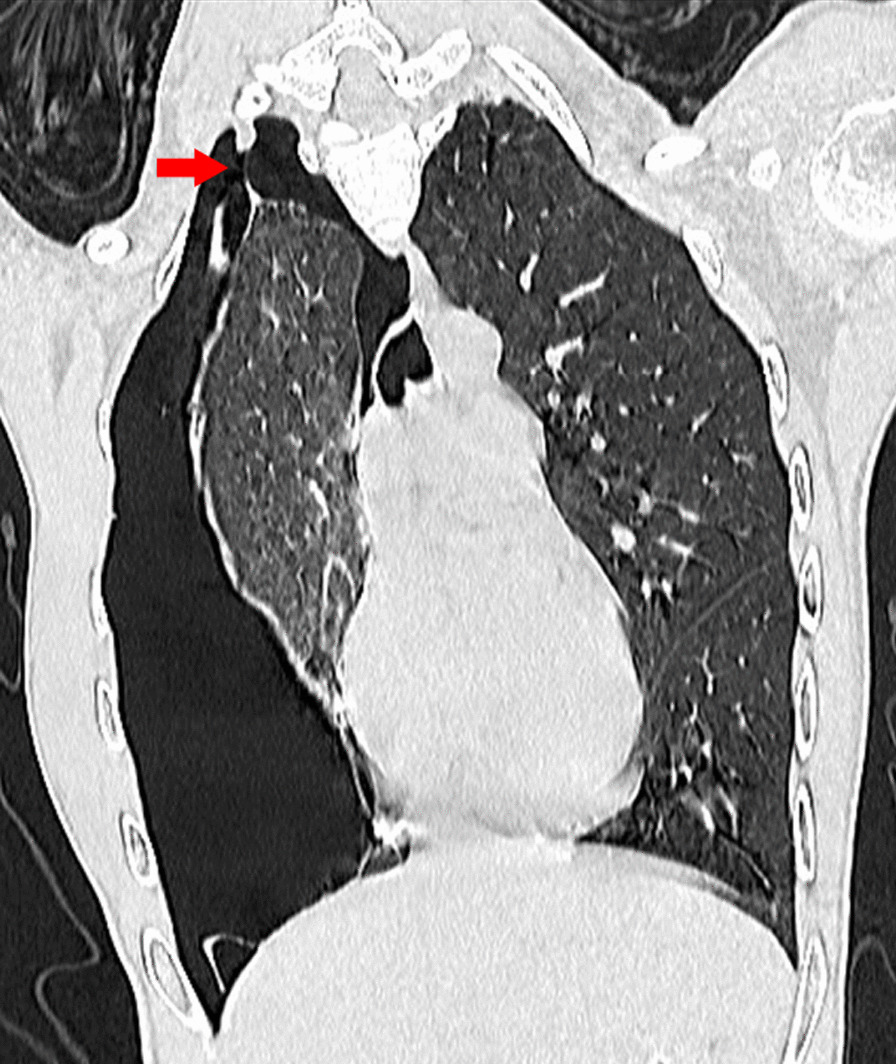
The coronal image shows the ruptured adhesion cord in the right pleural apex (red arrow)

At 10 h after localization, the patient complained of worsening chest pain while breathing and speaking, accompanied by chest distress, shortness of breath and cold sweats. The hemoglobin level of the patient was lower than 125 g/L, and an emergency chest CT scan (Fig. 2D) revealed a right-sided massive hydropneumothorax. A cord shadow could be seen at the right pleural apex (Fig. 3), which indicated ruptured pleural adhesions and a possible active bleeding point. Closed thoracic drainage therapy was then immediately performed, which led to the draining of incoagulable blood. The patient showed clinical signs of hemorrhagic shock such as pallor, cold extremities, low blood pressure, and soon became unconscious. Laboratory tests described significantly decreased hemoglobin level (87 g/L). Therefore, the resuscitation protocol for handling hypovolemic shock was performed to correct hypovolemia followed by rapid fluid infusion, blood transfusion and emergent thoracoscopic exploration.

**Fig. 4:**
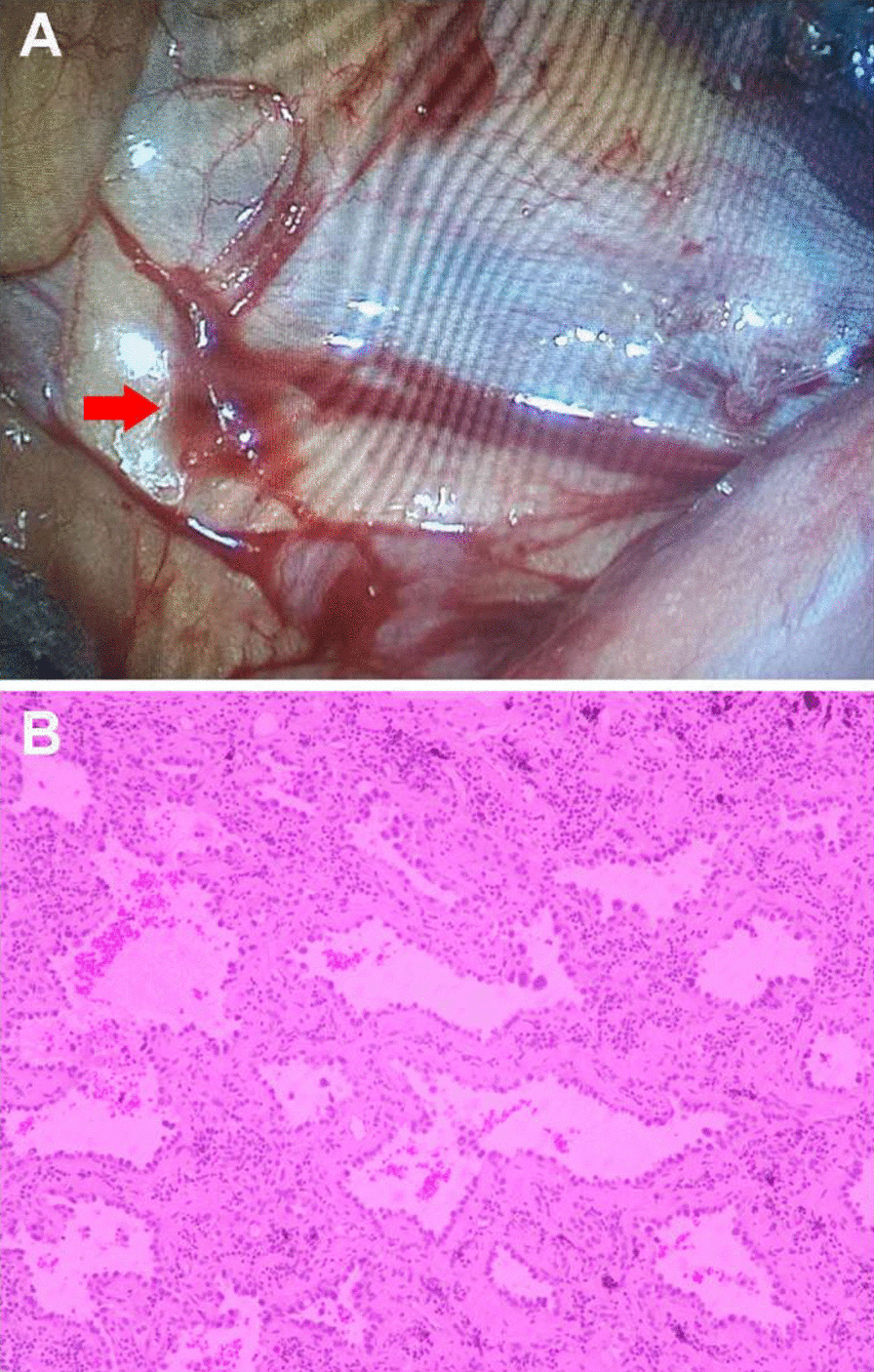
**A** The aberrant vessel (red arrow) in the right pleural apex under VATS. **B** Biopsy specimens were subjected to histopathology examination under a Nikon Eclipse Ci-S trinocular light microscope, which demonstrates the GGN in the right lung is a microinvasive adenocarcinoma (hematoxylin and eosin, 400 ×)

During the thoracoscopic exploration, no obvious bleeding was found at the localization puncture site. However, an aberrant vessel fractured and bleeding was discovered at the right pleural apex (Fig. 4A), which accompanied by blood clots and bloody fluid in the chest. We then performed electrocoagulation hemostasis for the fractured aberrant vessel. The GGN in the right lower lobe was then removed thoroughly by wedge resection based on the location of the microcoil. The excised nodule of approximately 0.5 cm in diameter showed ambiguous borders, and the biopsy demonstrated microinvasive adenocarcinoma (Fig. 4B). The patient recovered swiftly and was discharged from hospital 2 days after operation. The follow-up chest CT after 3 months showed surgical scars in the lower lobe of the right lung, and little effusion left in the right thoracic cavity. Laboratory tests indicated a hemoglobin level of 115 g/L.

## Discussion and conclusions

Microcoil localization surgery is generally considered a safe treatment option and usually does not cause severe postoperative complications [8]. However, pneumothorax has remained a common complication of localization surgery with a previous reported incidence of 9–67% [9, 10], and the incidence in our hospital is around 23% during the past 2 years (188 cases of pneumothorax out of 817 total cases), and when it comes to the GGNs in the lower lobes, the incidence is about 30% (159 cases of pneumothorax out of 538 lower lobe cases). To the best of our knowledge, this is the first case of postoperative patients with delayed massive hemopneumothorax and active hemorrhagic shock reported. In our case, it can be speculated that the progressive pneumothorax developed in the patient was caused by puncture injury.

After the localization surgery, a chest CT scan on the lesion level was conducted immediately and no signs of pneumothorax were found. However, the incomplete coverage of the thoracic CT scan could result in the neglection of early signs of pneumothorax. In our case, the coil was inserted by leaving-coil-end tail implantation method, which is simple to operate and greatly improves the efficiency of thoracoscopic surgery [11, 12]. VATS allows the surgeon to quickly locate the nodule based on the exposed tail on the parietal pleura, but the relative movement between the interface of visceral and parietal pleura with respiration can lead to further damage and increase the probability of pneumothorax [13, 14]. In this particular case, the localized coil was placed in the lower lobe with greater respiratory movements. Kha et al. reported another method of entire coil implantation, avoiding the damage caused by respiration movement, which may be a better choice for patients with pulmonary nodules in the lower lung lobes. However, it’s less efficient because it required the surgeon to find the localized coil by palpation during thoracoscopic surgery [15].

Pneumothorax alone is not enough to cause heavy bleeding. Rupture of aberrant vessels is one of the most common causes of active bleeding for spontaneous hemopneumothorax [16, 17]. In this case, the aberrant vessel in the pleural apex abnormally grows into the pleural cavity and attaches to the visceral pleura. With progressive pneumothorax, the lung collapsed and the pleural adhesions torn, which eventually resulted in the rapture of the the aberrant vessel. The emergency chest CT scan (Fig. 2D) and subsequent thoracoscopic surgery identified the aberrant vessel. Similar cases have been reported in spontaneous pneumothorax related pathologies [18, 19]. As in our case, the most frequent bleeding location is the superior thoracic aperture [18], possibly due to its higher incidence of pleural adhesions. It has been documented that the sphincter muscle is often missing in such aberrant vessels, so they usually have poor contractility and are more easily teared apart.

In our case, the aberrant blood vessel could be found from the chest CT scanned after hemopneumothorax, but not before. Although the incidence of aberrant vessel rupture is low, the consequences can be fatal. Therefore, during any procedure that may cause pneumothorax, operators should always be alert to complications like delayed massive hemopneumothorax, even hemorrhagic shock. Since conservative therapy with thoracic drainage could aggravate the bleeding, the thoracoscopic surgery should be performed immediately.

In addition, the time interval between the localization surgery and VATS is usually within 24 h. Due to the limited medical resources and other practical reasons, many patients could not undergo VATS on the same day of localization, and it was reported that more significant complications were not observed due to prolonged waiting time for surgery [20]. This is because microcoil localization is relatively safer and more reliable compared with hook wires, lipiodol, dye or other materials [21]. The microcoil is consider to be safely retained in the lungs of patients for a long time, which is not easily detached [22]. However, our case here suggests that the time interval between the two operations should be shortened as much as possible to reduce the incidence of complications. In a previous study, the researchers managed to shorten the time between CT-guided insertion and start of operation to 136.6 ± 89.0 min, and the complication rate is relatively low (5% of the intra-operative complication and 8% of the post-operative complication) [23]. In order to avoid significant complications like our case, now every patient in our hospital receives their VATS on the same day of the microcoil localization.

We performed routine blood pressure, oxygen, heart rate and electrocardiogram monitoring for 4 h for the patient after localization operation, and no abnormalities was detected during this time. The delayed hemopneumothorax occurred at about 10 h after localization surgery, thus we suggest that the monitoring period should be extended since the thoracoscopic surgery initiated. Once hemorrhagic shock occurs, the thoracoscopic exploration should be performed as soon as possible. Early thoracoscopic hemostasis is effective and does not affect the resection of pulmonary nodules. After this unfortunately incidence, every patient in our hospital received intense care and monitoring right until the time their VATS began.

In conclusion, the deadly complications are mainly caused by rupture of pleural aberrant vessels subsequent to puncture related pneumothorax. Shorten the time interval between localization and thoracoscopic surgery, extend the monitoring time after localization, and select a different localization approach for the lower lobe lesions might help to reduce the risk of these complications.

## Data Availability

All data generated or analysed during this study are included in this published article.

## Abbreviations

VATS: Video-assisted thoracoscopic surgery
CT: Computed tomography
GGN: Ground glass opacity nodules
